# A Treatise on Surgery

**Published:** 1888-12

**Authors:** 


### Reviews of Books.

---

*A Treatise on Surgery.* By T. HOLMES, M.A. Fifth Edition. Edited by T. PICKERING PICK, F.R.C.S. Pp. 1004. London: SMITH, ELDER, & CO. 1888.

A text-book which has reached a fifth edition in thirteen years ought to be good either on its own merits or by comparison with others. It is unfortunately the case in this country that the popularity of a text-book depends as much on its reputation for enabling students to pass examinations as on its real worth as a means of education. Thus, in surgery, it might happen that the work of a capable and honest teacher, reflecting the most advanced truth in his department, might be rendered nugatory simply because one or two men who stand at the examining portals have written a text-book which is either out of date or unsound.

With reference to the work now before us, we are bound to say that, as a means of educating the student, it is certainly inferior to every similar work in the English language. This is not saying it is altogether bad: simply that it is on the whole not so good as others. It would, indeed, be possible to go over the whole work, chapter by chapter, and show that, as an honest statement of the best surgery of the civilised world, it is deficient according to nearly every legitimate test. To do this would occupy far more space than we can spare; but it may be permitted us shortly to examine some of the sections which have been specially provided, or specially extended, for the fifth edition. These at least ought to be up to date and trustworthy.

The section on Diseases of the Bones and Joints has been specially added to. Redfern still does yeoman's duty in providing figures and ideas for the description of inflammation of cartilage. Brodie, too, and Rindfleisch are quoted. Now such names in a history of the advance of our knowledge of these diseases, for a large cyclopædia, deserve full recognition; but in a short summarised account of them, for a book like this, they ought to be replaced or supplemented by others whose influence on extending or completing our knowledge has been greater. Indeed, we really want no names at all, if the accounts provided are reflexes of our present knowledge; but if names are given, let them be representative.

The section on Intestinal Obstruction, which is also greatly added to, is somewhat colourless and vague, as if written by one who had studied a good work on the subject, but had failed completely to master it. In the Treatment of Obstruction, he says: "The first rule of practice is to abstain from irritating the bowels with purgatives, to give nourishment in the fluid form only, and in the smallest possible quantities at a time, and to soothe the patient's suffering with opium, subduing thirst with small quantities of ice kept in the mouth." Venturing to call these *rules* of practice, we would remark that there is only one, the first—to abstain from purgatives—to which objection could not be taken. The administration of opium in any form of intestinal obstruction is undoubtedly a bad "*rule* of practice." The objections to it are so well known, and have been so frequently urged, that the most casual student must open his eyes at this "rule of practice" provided in our latest surgical text-book. As to the second rule, it might be pointed out that nourishment of any sort by the mouth simply lies unabsorbed by the stomach, and is returned with the other unabsorbed fluids. Rarely opium may be given, and sometimes food by the mouth; but not as "rules of practice." Not a word is said of enemata, the most valuable, if not the only, means of giving nourishment. And so it is all through. The writer has failed to grasp the most important points—a fatal fault in the writer of a text-book.

Of the whole chapter on Surgical Diseases of the Female Organs of Generation, nothing better can be said than that it is bad. In some cases it would seem as if names of operators were omitted with deliberate intention. Thus the description of the surgery of ruptured perineum "facilitated by an assistant putting his forefinger in the bowel" *(sic)* completely ignores the operations of such technical masters as Tait and Bantock, and provides a by no means efficient substitute. The operation of hysterectomy for fibroids is described: in the only point in which it is definite it is almo st ridiculous—thus: "Before the wire (of the serre-nœud) is tightened it must be carefully examined to ascertain that it does not include the fundus of the bladder or a loop of small intestine." A man who is likely unwittingly to include a loop of intestine in the wire clamp, and leave it there till it is tightened, had better leave abdominal surgery alone.

"The ovary is liable to tumours of all kinds." Is it indeed? If we do personify that important organ (and there is precedent for it), we might truthfully say that She (feminine by preference) is liable to undergo misrepresentations, mutilation, and even complete detachment from the sources of life; but to be "liable to tumours of all kinds" is surely too heavy an indictment. "The fibroid tumours are at first difficult to distinguish from the cancerous, but the different rate of growth will settle the question ultimately." In the complete disregard of fact and experience this sentence approaches the sublime. Fibroid tumours are spoken of as if their diagnosis from cancers were a matter of every-day occurrence. Does the writer know how many times a fibroid tumour of the ovary has been found? Does he know that it is among the rarest of all growths? The "rate of growth will settle the question ultimately": we should think the death of the patient would be an earlier ultimate settling of the question. "No surgical interference is advisable in solid tumours of the ovary." Now a medical student who makes a statement like this should either be relegated to his studies, or be made to give very full reasons for going in the teeth of the opinions of those best able to judge.

The description of Ovariotomy is somewhat remarkable. It begins thus: "Ovariotomy is thus performed. The patient should have been well purged, and should have her legs covered with a pair of warm drawers. The room should be warm—nearly 70°. A large band is to be passed round the belly, of waterproof cloth with a hiatus for the incision. She should be in the recumbent position, on a firm table." How like to the examination-paper of a doubtful candidate is this! The passing round the belly of the well-purged and bedrawered patient of a band of waterproof cloth is followed by laying her in the recumbent position on a firm table! Has anyone, by the way, suggested that the operation be performed in the sitting position on a ricketty arm-chair? "An incision is made in the linea alba from a little below the umbilicus to a little above the pubes." A nice gash this through which to begin the removal of a big but simple ovarian cyst! "The surgeon introduces his fore and middle fingers, and sweeps them round over the cyst to ascertain in the first place if he is really in the peritoneal cavity (!), and secondly to feel for adhesions. These, if present, are gently separated from the wall of the cyst until the whole hand is introduced, and the cyst is freed from adhesions on all sides." If this is the practice of the author, he ought at least to have inserted a footnote to explain that it differs from that of all the best operators. Here, as elsewhere, the work and advice of competent teachers seems to be ignored in a manner that appears as if it were studied.

Take the treatment of the pedicle, for instance. "Three ways are in use for this purpose: viz., to fix a clamp on the pedicle outside the wound; to divide the pedicle with an ecraseur, cauterise it if necessary, and drop it back into the pelvis; or, to tie the pedicle with carbolised silk, cut the ligature short, and remove the tumour." Now it is surely needless to say to any even moderately educated man that the first two ways are *not* in use; and of the third way, that it would be true if, instead of "carbolised silk," we read "silk or catgut." The ecraseur and cautery for ovariotomy may be the substitute of a London surgeon for the method (by clamp, not ecraseur) of a certain individual named Thomas Keith, who is not unknown in the history of ovariotomy, but this substitute can scarcely be spoken of as a "way in use." Keith's method is not even recognised as being "in use"; but "the ecraseur and cautery if necessary" is put down glibly as one of the three recognised methods.

But we have lost patience with the whole article. The grandest operation of modern surgery was probably never more unworthily presented to a reading public. If this article has been written or edited by a "Member of the Court of Examiners of the Royal College of Surgeons of England" (as is said on the title-page), then teachers who try to place before their students the best surgical practice of the year of grace in which we live will have a desperate list of rejections among their pupils.

We heartily admit the difficulty which one or even two men must have in writing a book on surgery which will satisfy all critics. In America several authors have approached perfection as nearly as, in all probability, single men ever will; at least two in England have written admirable text-books; as to the work before us, while we gladly admit that much of it is good, we must say that not a little is indifferent, and that too much is distinctly bad.

